# Kenya’s Health in All Policies strategy: a policy analysis using Kingdon’s multiple streams

**DOI:** 10.1186/s12961-019-0416-3

**Published:** 2019-02-06

**Authors:** Joy Mauti, Lara Gautier, Jan-Walter De Neve, Claudia Beiersmann, Jale Tosun, Albrecht Jahn

**Affiliations:** 1 Heidelberg Institute of Global Health, Heidelberg University, Im Neuenheimer Feld 130.3, 69120 Heidelberg, Germany; 2 Department of Social and Preventive Medicine, School of Public Health (ESPUM), University of Montreal, 7101, avenue du Parc, 3rd floor, Montreal, Quebec H3N 1X9 Canada; 3 Centre d’Etudes en Sciences Sociales sur les Mondes Africains, Américains et Asiatiques (CESSMA), Sorbonne Paris Cité University, Case courrier 7017, 75205 Paris Cedex 13, France; 4 Institute of Political Science, Heidelberg University, Im Neuenheimer Feld 130.3, 69120 Heidelberg, Germany

**Keywords:** Health in all policies, intersectoral collaboration, agenda-setting, social determinants of health, Kenya

## Abstract

**Background:**

Health in All Policies (HiAP) is an intersectoral approach that facilitates decision-making among policy-makers to maximise positive health impacts of other public policies. Kenya, as a member of WHO, has committed to adopting HiAP, which has been included in the Kenya Health Policy for the period 2014–2030. This study aims to assess the extent to which this commitment is being translated into the process of governmental policy-making and supported by international development partners as well as non-state actors.

**Methods:**

To examine HiAP in Kenya, a qualitative case study was performed, including a review of relevant policy documents. Furthermore, 40 key informants with diverse backgrounds (government, UN agencies, development agencies, civil society) were interviewed. Analysis was carried out using the main dimensions of Kingdon’s Multiple Streams Approach (problems, policy, politics).

**Results:**

Kenya is facing major health challenges that are influenced by various social determinants, but the implementation of intersectoral action focusing on health promotion is still arbitrary. On the policy level, little is known about HiAP in other government ministries. Many health-related collaborations exist under the concept of intersectoral collaboration, which is prominent in the country’s development framework – Vision 2030 – but with no specific reference to HiAP. Under the political stream, the study highlights that political commitment from the highest office would facilitate mainstreaming the HiAP strategy, e.g. by setting up a department under the President’s Office. The budgeting process and planning for the Sustainable Development Goals were found to be potential windows of opportunity.

**Conclusion:**

While HiAP is being adopted as policy in Kenya, it is still perceived by many stakeholders as the business of the health sector, rather than a policy for the whole government and beyond. Kenya’s Vision 2030 should use HiAP to foster progress in all sectors with health promotion as an explicit goal.

## Background

Health in All Policies (HiAP) is an emerging strategy for governance in health in low- and middle-income countries (LMICs); it aims to ensure that the social determinants of health (SDH) are taken into account by other development sectors [1, 2]. At the 55th Session of the Regional Committee of the African Ministers for Health in 2005, the ministers commissioned the Regional Director to prepare a strategy for Africa to address SDH [3]. This resulted in the African region endorsing a regional framework for “*tackling health inequalities and improving the health of the poor and vulnerable through action on the social determinants of health*” [4]. Eight years later, in 2013, the WHO Regional Office for Africa (AFRO) led discussions on how HiAP can be implemented at national level in African countries. The discussions revolved around a draft analytic framework for HiAP and intersectoral collaboration (ISC) [4, 5]. In this meeting, WHO AFRO also drafted a regional position statement, highlighting key principles for implementing HiAP in the African context [4].

As a WHO member state, Kenya has committed to the HiAP approach and has explicitly featured the approach as objective 6 in the national ‘Health Policy’ for the period 2014–2030 [6]. It has also stated that, through the HiAP approach, the country will seek to demonstrate a ‘win-win’ or ‘co-benefit’ situation for all sectors and all stakeholders across the board [6, 7]. In 2017, at the World Medical Association Council meeting in Livingstone, Zambia, Kenya made a commitment, alongside other African countries, to set up intersectoral collaborative groups [8]. Beyond its health policy, Kenya has an overall national development framework called Vision 2030 [9, 10]. This framework is the blueprint to be a middle-income country by 2030, addressing the social, economic and political components of development in order to further facilitate Kenya’s progress towards the global development goals – the previous Millennium Development Goals (MDGs) and the current Sustainable Development Goals (SDGs) [9, 11].

This empirical qualitative research study investigates how the HiAP approach made it to the political agenda in Kenya, and to which degree the commitment to HiAP is formulated in policy. The study highlights pertinent issues that inform stakeholders and academia in Kenya and other developing countries in the process of adopting HiAP.

### Conceptual framework: problem, policy and politics

Leppo et al. [12] and Stahl et al. [2], among others, have shown that HiAP is underpinned by the Kingdon framework. As such, to evaluate adoption of HiAP in Kenya, the study draws upon Kingdon’s multiple streams model [13, 14]. The multiple streams framework postulates that policies are made when the problem, politics and policy streams converge resulting in a ‘policy window’. The ‘problem stream’ looks at how situations rise to the agenda using indicators that are used to monitor changes of the situation, for example, reports or evaluations [13, 15]. The ‘policy stream’ describes which policy initiatives and solutions are being proposed and developed [13, 15]. The ‘politics stream’ indicates how the government and national mood as well as campaigns by interest groups can influence whether or not an idea rises to the agenda [13, 15]. This process is propagated by the ‘policy entrepreneurs’. Policy entrepreneurs are individuals whose timely and strategic actions influence which policy rises to agenda-setting, the policy process and its outcomes [13, 16]. These actors are catalysts in the coupling of the various streams to create a window of opportunity advancing the policy [13, 17]. We examined the three streams in the context of HiAP in Kenya:

1. Problem stream – What problems led to the HiAP being sought after as a policy solution in Kenya?
2. Policy stream – How has HiAP been/is HiAP being adopted in Kenya?
3. Politics stream – What are the political factors for or against the adoption of HiAP in Kenya?

The study also reviews two essential ingredients in Kingdon’s framework, namely (1) policy entrepreneurs – who were/are the champions of change advocating for HiAP? and (2) window of opportunity – how do these streams interplay to create a window of opportunity?

## Methods

### Study setting

Kenya is a country located in East Africa with a population of approximately 49 million people in 2017 as per World Bank data [18]. Following the 2013 constitution referendum, Kenya transferred decision-making power, resources and representation from centre (national) to local (county) levels, i.e. devolved government [19–21]. Kenya now has 47 semi-autonomous county governments and associated structures such as county treasury and assemblies [10, 20]. Given that the counties governance structures have not yet been fully established and governors change every 5 years, it is expected that this constitutional change will have an implication on ISC.

In this new structure, the Ministry of Health in the central government still holds the policy-making and regulation mandate. The county governments are mandated with policy implementation as well as management of resources [19]. With the guidance of the national Ministry of Health, the respective county health departments or selected committee will need to play a central role to streamline HiAP in their given setting [3]. Public sector financing has also remained constant over the last decade, at approximately 29% of total health expenditure, whereas donors’ contribution has more than doubled, increasing from 16% in 2001/2002 to 35% in 2009/2010 [6]. The funds are directly channelled from central treasury to the counties who solely decide how the funds are spent at county level. Donors can also directly fund projects within a given county, independent of the government contributing to external financing, which is off budget. These reasons pose a challenge for any policy formulation and can have implications for national ownership of the HiAP agenda. Table 1 highlights some key country indicators, while Table 2 lists key policies related to HiAP adoption in Kenya.

**Table 1:** Kenya country indicators

Indicators/Year	2000	2010	2017
Population, total (millions)	23.4	31.45	49.7
Population growth rate (%)	3.3	2.7	2.5
Life expectancy at birth, both sexes, total (years)	58	63	67
GDP growth, annual (%)	0.6	8.4	4.9
Unemployment rate (%)	10.1	12.01	11.47
School enrolment, primary and secondary (gross), gender parity index	1	1	n/a

**Table 2:** Key policies linked to HiAP in Kenya

Year	Policy
2005	‘*Tackling health inequalities and improving the health of the poor and vulnerable through action on the social determinants of health*’ Framework
2008	Vision 2030
2010	The constitution of Kenya – Decentralisation
2011	Kenya Health Policy 2014–2030 – HiAP as a policy objective
2013	WHO AFRO regional statement

### Study design

This is a qualitative case study to examine the adoption of HiAP in Kenya using extensive document review and semi-structured interviews with key informants.

#### Document review

Our document review encompassed both peer-reviewed publications and grey literature and included the following steps. First, the PubMed database was searched to retrieve peer-reviewed publications on this topic using a combination of keywords related to HiAP, including “multisectoral action” and “intersectoral collaboration”. Second, searches were conducted on ministry government websites that were accessible on the internet. On Google Scholar, an additional search using “HiAP and Kenya” as keywords for grey literature was conducted. We also included major articles and documents previously known to the study team. Notably, the Health Policy document for Kenya for the period 2014–2030 was the first document to be assessed to identify how the government described HiAP through the Ministry of Health in Kenya context. Through searching online grey literature, one report on HiAP, the outcome of one researcher’s work, was identified. [22] At the health promotion unit office under the Ministry of Health, a draft policy document for HiAP framework in Kenya was identified. At the WHO office in Kenya a report evaluating SDH in Kenya was also retrieved [23].

#### Data collection through interviews

A purposeful sampling approach was used to select the first batch of key informants, followed by a snowball approach (e.g. first key informants providing additional names of resource persons whom we also included in the list of potential key informants) to reach data saturation. We purposefully approached two types of respondents in each ministry: working at the highest level of policy-making and working at technical level. We also sought to include informants from relevant categories who had been involved in the policy process, e.g. bilateral cooperation agencies, civil society, academia, independent consultants and policy institutes. Informants were contacted by walking into their offices, phone or email and, based on their approval to participate, were recruited by the interviewer (first author) to data collection.

The questions focused mainly on the knowledge of HiAP, the interviewee’s personal contribution or interaction with HiAP, HiAP in policy documents, the perception of ISC, and the role of HiAP in Kenya in relation to the pursuit of global development goals, especially the SDGs. Written consent to record the interviews was sought in all cases. Interviews generally took place at the interviewees’ work place. All interviews were conducted in English and recorded, with the exception of four interviews, as the study participants did not consent to being recorded. Notes were conscientiously taken throughout all interviews.

In total, 40 in-depth interviews with various stakeholders were conducted. In Table 3, we show selected characteristics of the study participants. The informants’ profiles ranged from government officials, development partners, implementing partners (NGOs – consortia and grassroot) to independent consultants and academic professors in Kenya. The government key informants were all from national level. The specific titles of the range of positions held were Under-Secretary, Chief Economists, Deputy Economists, Economists, Policy Director, Deputy Policy Director, Architects/Housing Officer, Head of Departments and department members. Sixteen of the 20 contacted ministries in 2016/2017 participated in the study. Interviews with two ministry representatives (from the Ministry of Mining and Ministry of Information and Technology) could not be conducted due to technical issues, and two ministries (Ministry of Defense and Interior Coordination of National Government and Ministry of Tourism) declined. The authors intended to achieve gender balance in this study. However, all the positions of interest within the study period were predominately filled by men; hence, only 10% of the interviewees were female.

**Table 3:** Selected characteristics of Kenyan ministry, development and non-governmental interviewees (2016–2017)

	Interviewed
Number of Interviewees – Government	
Ministry of Devolution and Planning	2
Ministry of Finance and National Treasury	1
Ministry of Foreign Affairs and International Trade	1
Ministry of Education	1
Ministry of Health	3
Ministry of Transport and Infrastructure	4
Ministry of Environment, and Natural Resource	1
Ministry of Land, Housing and Urban Development	2
Ministry of Sports, Culture and the Arts	1
Ministry of Labour and East Africa Affairs	1
Ministry of Energy and Petroleum	1
Ministry of Agriculture, Livestock and Fisheries	3
Ministry of Industrialization and Enterprise Development	1
Ministry of Public Service, Youth and Gender Affairs	1
Ministry of Water and Irrigation	1
	24
Number of Interviewees – Development Partners	
WHO – Kenya Office	1
IOM – Kenya	1
GIZ – Kenya	1
World Bank – Kenya	1
International NGO	2
	6
Number of interviewees – non-government	
NGO – Consortiums	2
Grassroot NGO	1
Academia and Policy Analysis Institute	5
Independent Consultants	2
	10
TOTAL	40
Range of positions	
Undersecretary	1
Chief economist/deputy chief economist/economist	8
Architects/housing officer	2
Director-policy/deputy director-policy/policy analyst/statistician	9
Head of NGOs/country directors/programme officers/coordinators	7
Head of departments	3
Department members	5
Academic professor/lecturer	3
Independent consultant	2
Gender (Female %)	4 (10%)

*GIZ* (Translated into English) German Corporation for International Cooperation, *IOM* International Organization for Migration, *NGO* non-governmental organisation

### Analytical approach

A qualitative framework analysis was used to analyse the interviews as well as the sections addressing health in the policy documents [24, 25]. All interviews were transcribed and uploaded into NVivo. Selected sections of policy documents were also highlighted in NVivo. As summarised in Table 4, the codes and nodes were used to capture the interviewees’ answers and policy document information. They were then assigned under the themes ‘problem’, ‘policy’ and ‘politics’, which are the Kingdon model streams being used in this study [26].

**Table 4:** A summary of nodes under the three streams of the Kingdon framework

	Problem	Policy	Politics
Nodes	• Social determinants of health challenges • Knowledge of HiAP • Knowledge of intersectoral collaboration	• HiAP in policy documents • Specific contribution by interviewee to HiAP • Millenium Developmen Goals and HiAP • Vision 2030 and HiAP	• Intersectoral bodies and processes • Intersectoral collaboration as present/absent/not serious • Role and collaboration of specific ministries/examples of potential HiAP collaborations

## Results

The results are presented along the problem stream, policy stream and politics stream. Where applicable, data from documents will be reported first followed by that of interviews.

### Problem stream

The problem stream highlights some of the major health challenges and issues of how they are being addressed.

#### The double burden of diseases calling for a SDH approach

Over the years, Kenya has sought interventions to address key health challenges, such as maternal and child health and nutrition, HIV/AIDS and tuberculosis, malaria, and the emerging threat of non-communicable diseases, with mixed results. The SDH which Kenya intends to address in the period 2014–2030 include access to safe water and adequate sanitation, nutrition, safe housing, occupational hazards, road safety, security, income, and community participation [6]. Even though the influence of the social and economic determinants on the key health challenges is increasingly being recognised, some interviewees thought the recognition among stakeholders is not enough.

“*The notion of social determinants and underlying determinants hasn’t been around for a very long time… It is like if you are doing what you are doing, it is like you are okay, let’s come together it will work. But understanding the need to address the cause of the causes, they still have a long way to go.*” (KI13).

Addressing SDH in the context of HiAP was specifically mentioned by seven of the interviewees. One interviewee mentioned SDH in the context of health literacy at community level.

#### Lack of knowledge of global policies at national and local level

While HiAP is promoted as a specific policy option to tackle the SDH first at international level, most interviewees indicated that there is a prevailing lack of knowledge of global policies and declarations on the ground.

“*…first of all they don’t know about this and I can tell you that even people in the Ministry of Health and people in county governments take a long time before they become aware of these international decisions and their implications… I have worked with development for so long, many policies are changed even before they are known.*” (KI2)

Outside the Ministry of Health, no other ministry official interviewed in this study had ever heard of “Health in All Policies” as a term, but they were familiar with ISC as a concept. Table 5 compares the knowledge of ISC versus HiAP among key informants.

**Table 5:** Knowlegde of intersectoral collaboration (ISC) versus Health in All Policies (HiAP) among the various stakeholders

Sector	Total number of informants	Knowledge of ISC only	Knowledge of both HiAP and ISC
Government Ministries	24	21	3
Development partners	6	4	2
NGOs	3	2	1
Academia	5	–	5
Independent consultants	2	–	2

### Policy stream

The policy stream highlights how competing solutions and existing frameworks influenced the adoption of an effective HiAP approach.

#### HiAP in the health policy documents

Indeed, the HiAP approach as a critical solution to address complex health issues was specifically mentioned in international resolutions such as the World Health Assembly resolution WHA67.12, to which Kenya is committed. The Kenya Health Policy 2014–2030 aims to attain the highest standard of health in a manner responsive to the needs of the Kenyan population. HiAP has been specifically outlined in this policy document as Policy objective 6 and multi-sector action for health is outlined as policy principle number 3.3.4.

Two reports from studies advocated by WHO Kenya evaluating the potential HiAP and the status of SDH in Kenya emphasised the need for its adoption [22, 23]. The SDH status report showed the importance of involving not only the government agencies but also the non-governmental actors as well as the community in addressing health. From these two reports, the WHO Kenya country office helped the health promotion unit to draft a HiAP framework; this is the unit mandated to spearhead HiAP adoption in Kenya. This draft policy document had not yet been endorsed by the government at the time of the study. Based on this draft HiAP document and the SDH report, Table 6 shows how SDH can be associated with the sectors and issues outlined in the Adelaide Statement on HiAP. It also shows some outcome indicators highlighted in a previous unpublished study evaluating SDH in Kenya and examples of their potential impact on health [3, 23, 27]. Table 7 highlights the many roles to be played by the various stakeholders as presented in the Health Promotion Strategy for Kenya.

**Table 6:** Health in All Policies in Kenya: social determinants of health and health impact indicators

	Sectors according to Adelaide Statement	Social determinant of health	Outcome indicators	Health impact indicators (examples)
1	Education and early life	Women’s literacy	Literacy level	Contraceptive prevalence Maternal mortality ratio Assisted delivery Access to specialised skilled health services Control of environmental degradation Life expectancy Neonatal infant and child mortality
2	Environment and sustainability	Access to safe water	Quality of water and air	
3	Agriculture and food	Adequate nutrition	Proportion of households reporting food insecurity Affordability and availability of nutritious food	
4	Housing and community services	Safe housing	Quantity and quality of housing	
5	Economy and employment	Occupational hazards, unemployment	Unemployment rate Proportion (%) of households below poverty	
6	Infrastructure and planning, and transport	Road safety	Means of transport, road distribution (% of paved roads)	

*Notes:* Sources include the Adelaide statement on HiAP (column 1). This is the outcome report from the HiAP meeting in Adelaide showing how to engage policy-makers in HiAP, Kenya Health Policy document 2014–2030 (column 2) Wangombe et al. [23], HiAP framework draft document (column 3)

**Table 7:** Outlined roles of key actors in health promotion as listed in the Health Promotion Strategy for Kenya [44]

ACTOR	ROLE
Ministry of Health Headquarters (Health Promotion Unit, HPU)	◦ Policy formulation and review ◦ Setting strategic plan for the HPU ◦ Operational research ◦ Human resource development ◦ Standards and guidelines setting ◦ Engagement of partners and private sector ◦ Advocating for public health policies ◦ Monitoring and evaluation of health promotion activities/programmes ◦ Coordination, development and implementation of this strategy
County government	◦ Allocate funding for the strategy implementation ◦ Support allocation of funds for implementation of health promotion (HP) policy documents by the county government ◦ Support use of the documents by all HP practitioners ◦ Support the use of HP website for health information to empower communities take control of their health ◦ Support funding of communication activities
Other government ministries, institutions	◦ Adopt the Health in All Policies approach ◦ Develop and implement HP interventions with support from HPU ◦ Participate in the implementation and review of this strategy
Community and selected population groups	◦ Take part in planning and decision-making about health development programmes ◦ Engage in healthy behaviour ◦ Form partnerships with government and other key players in health promotion to effectively address the determinants of health
Development partners	◦ Support the strategy development and implementation processes ◦ Adopt recommendations/interventions of the HP ◦ Support training of HP staff ◦ Support development of standards, guidelines, framework and other tools as indicated in this strategy
Training and academic institutions	◦ Incorporate a competency-based approach to HP course development and implementation ◦ Develop curricula as suggested in the strategy and use them for training purposes ◦ Support development of evidence on HP effectiveness through research, monitoring and evaluation

#### Some interviewees’ specific contribution to HiAP

In total, nine interviewees had specifically contributed to developing the HiAP strategy in Kenya. Two of these nine interviewees participated in the discussions of HiAP during the 8th Global Conference of Health Promotion in Helsinki, Finland, on behalf of Kenya. They acted as representatives of Kenya to WHO. One of the independent consultants who had worked at the WHO headquarters and came back to Kenya indicated that they were part of the team in Geneva that was mandated with the initial critique of all the major papers submitted from all over the world to inform the HiAP document. When they returned to Kenya, the interviewees had several meetings planned by the WHO country office attended primarily by the Ministry of Health staff to ensure that HiAP was understood and was being supported. They led the team that prepared the draft HiAP framework. Some interviewees were also part of this team; however, when the draft document for the HiAP framework was shown to them, they said they remembered the meetings they attended and the drafting process, but it was the first time they were seeing that document. This showed that, once it was drafted, not much further action was taken.

“*We put the cart before the horse. We developed the health promotion policy without a clear way for implementing. We have not really started working on Health in All Policies.*” (KI11)

On the civil society’s side, a local NGO alliance also held a series of workshops in the Kenyan town of Kitale to bring these global policies to the local people.

“*…you know, so we bring the information from these conferences and share with the communities for the communities to interpret those global policies and discuss how they relate to them and how they can implement them. So, in fact, health in all policies is one of the directions or policies that we came with from Helsinki to Kitale…*” (KI2)

#### HiAP in Kenya in relation to development goals

The MDGs were adopted to address the major health challenges (i.e. maternal and child health, and the three major infectious diseases). MDGs informed Kenya’s national development framework Vision 2030. Vision 2030 is the long-term development blueprint for the country, aiming to transform Kenya into a “*globally competitive and prosperous and newly industrialized middle-income country providing a high quality of life to all its citizens in a clean and secure environment by 2030*” [9]. Therefore, Vision 2030 sets a platform for ISC but neither specifically mentions HiAP as a strategy nor has an emphasis on addressing the SDH.

With the adoption of SDGs in 2016, Vision 2030 was also modified to address them. Each of the 17 SDGs was mapped with Vision 2030 Second Medium Term Plan objectives to ensure the global development framework and its implementation is directly linked towards achieving both Vision 2030 and the SDGs. Several interviewees believe that the SDGs will be a great opportunity to foster ISC.

“*Well I don’t want to pre-empt but if there has been a time when we really need dialogue on intersectoral collaboration it is now because we are now phasing in SDGs at the same time we have had the opportunity to evaluate ourselves on the performance of the SDGs so we are better placed now to take opportunity of the SDGs and deliver value by talking about intersectoral collaboration now cause in the health sector intersectoral collaboration has been identified as a gap, yes.*” (KI32)

### Politics

HiAP as a policy approach is influenced by the political climate and government structure of a country. The Kenyan Constitution is the overarching legal framework to ensure that every person has a right to health [10]. The government structure presents a unique challenge for HiAP, as illustrated in the next section.

#### Intersectoral bodies and process

The main institutional structures of Kenya’s Vision 2030 are the Vision 2030 Delivery Secretariat and the Vision 2030 Vision Delivery Board. The Vision Delivery Board consists of both government officials who are permanent secretaries in the various ministries and private sector. They operate through a Presidential Order Gazette Notice No. 1386 of February 2009. The Vision 2030 Delivery Secretariat ensures that Vision 2030 flagship projects are incorporated in the various ministries’ performance contracting targets. Table 8 shows the various sectors in the given pillars.

**Table 8:** An outline of the various sectors in Kenya under the Vision 2030 Pillars

Vision 2030 Pillar	Sectors
Economic and Macro Pillar	• Tourism • Agriculture and Livestock • Wholesale and Retail, Trade • Manufacturing • Financial Services • Business Process Offshoring • IT-Enabled Services
Social Pillar	• Education and Training • Health • Water and Sanitation, Environment • Housing and Urbanisation • Gender • Youth • Sports and Culture
Economic Pillar	• Decentralisation • Devolution • Governance and rule of Law

Source: Vision 2030 website (https://vision2030.go.ke/, data accessed and retrieved in June 2018)

However, more than half of the interviewees still indicated that there is no substantial collaboration between the ministries. Additionally, meetings are held only on an as-needed basis and not regularly. Most of the government interviewees indicated that collaboration between sectors occurred mostly at budget planning and reporting stages. However, ISC to address health problems that span multiple sectors is thus far quite arbitrary.

“*There are committees, I just know them as… but really, okay, I have also worked in parliament and I only know of the specific committee that is on health, committee on security, committee on human rights, like that. But as in having the committees, like the representatives from various thematic areas coming together to sit just so that they can do health, I am waiting to hear that (laughs).*” (KI16)

Others showed that previous intersectoral committees also consisting of permanent secretaries with a focus on SDH were no longer active.

“*Under the auspice of social determinants of health, during that time, there used to be the national economic and social committee, where our permanent secretaries used to meet used to be called NESC, where they used to discuss as sectors and we know from such committees that there was lot of discussion on how the different sectors can work together… It is not there anymore I think it died.*” (KI14)

One interviewee thought that, for HiAP to work in Kenya, it should be hosted in a “*super department*” such as the office of the president.

“*But now when it comes to having ministries meeting across, for example, minister for education cannot call the CS* [Cabinet Secretary – equivalent to minister] *for health. As who, you understand? Because that is separate, it is only the president now who can do that and at county level, it is only the governor who can do that*.” (KI15)

St-Pierre et al. [28] discuss the nature of governance tools for HiAP. Table 9 outlines some tools already present for HiAP for Kenya, and also shows some which HiAP in Kenya can make use of.

**Table 9:** Present and potential governance tools for HiAP in Kenya

Nature	Tools	Present or Potential for HiAP
Structure	Cabinet Committees/interdepartmental committee	The Apex and Vision 2030 Delivery Boards (potential)
	Dedicated organisations/units	Health Promotion Unit (present)
Process	Planning and priority-setting processes	Budgeting process (potential)
Mandate	Agreement protocol	Health policy 2014–2030 (present)
		The draft HiAP framework (present)

All government interviewees indicated that they did collaborate with development partners, especially for finances and technical capacity, and the civil society for implementation and advocacy as specific ministries. Table 10 summarises examples of areas on which the interviewees indicated that they collaborated or could collaborate with the Ministry of Health.

**Table 10:** Present and potential examples of collaboration mentioned by key informants

Ministry	Examples of collaboration
Ministry of Transport and Infrastructure	Ambulances, HIV awareness along road construction sites (present) *Boda boda* (motorcycle) transport (potential)
Ministry of Education	Immunisation, Deworming, Birth certificate (present) School of Health Policy (present)
Ministry of Agriculture, Livestock and Fisheries	Feeding programmes, Funding farmers (present) Nutrition Policy (present)
Ministry of Water and Irrigation	Cholera outbreaks (present)
Ministry of Environment and Natural Resources	Sewage treatment (present) Recycling plants (present)
Ministry of Land, Housing and Urban Development	Slum upgrade (present)
Ministry of Devolution Planning	Salary schemes for health personnel at county level, county placements and transfers (potential)

Most government officials indicated that the main health issues for which they collaborated with the Ministry of Health were HIV/AIDS and occupational health and safety. This was especially the case after the president declared HIV/AIDS a national disaster. Every ministry has an HIV unit or officials that deal with HIV-related issues. While there appeared to be attention to health issues such as HIV across sectors and to some extent ISC to address a number of health issues, a lot more effort was required.

“*I think Kenya has always mainstreamed the issues of health and no wonder the concept of health in all policies has not been so pronounced in Kenya, particularly in this ministry, that is why I am telling I have only heard it from you but it is because the issues of health are being addressed somehow, yes, in whatever we are doing.*” (KI30)

### Window of opportunity and policy entrepreneurs

A window of opportunity was created once HiAP gained enough political attention as a strategy to address the SDH in efforts to combat the double burden of disease. WHO Kenya office has propagated efforts to map the SDH in Kenya and HiAP adoption by collaborating with the ministry and academia, among other stakeholders.

Preparation and participation in global conferences and initiatives have represented a window of opportunity for HiAP. Kenya was part of a special study on SDH consisting of a total of 11 countries (least developed, developing and developed countries) from Africa. Each country was to analyse its national capacities for addressing the SDH at ministry level in collaboration with academic and research institutions.

A focus on SDH in Kenya was championed by a former Minister of Health (2005), Hon. Charity Ngilu, who was among the 19 Commissioners in the global Commission for Social Determinants of Health [3]. The draft HiAP document points out her work in addressing SDH in Kenya. Only one interviewee mentioned her as a policy entrepreneur. Unfortunately, the ministers after her did not propagate for HiAP with the same tenacity. The other nine interviewees who contributed directly to HiAP also acted as policy entrepreneurs, playing a key role in bringing HiAP to Kenya. Currently, the main policy entrepreneur for HiAP is the WHO country office in Kenya.

## Discussion

This study aimed to investigate how HiAP rose to the agenda-setting level and its adoption in Kenya using the Kingdon framework. The key findings revealed that some of the problems include the lack or little knowledge of HiAP among the stakeholders outside the health sector. There was more focus on ISC under the Vision 2030, in which HiAP is not an explicit strategy. The study highlighted how HiAP could foster development and the need for political commitment from the highest office in the country. The discussion will focus on these issues.

A key concern is that ISC is still quite arbitrary and much less a focus on health. The Vision 2030 that translated the MDGs to the Kenyan context did not have HiAP or intersectoral action as an explicit goal. The 9th Global Conference on Health Promotion, co-organised by WHO and the National Health and Family Planning Commission in November 2016 in Shanghai, China, reiterated the interconnectedness of health and all the 17 SDGs, calling for a political choice for health to move beyond fragmentation to strengthened policy coherence and efficiencies for improved health, health equity and development.

Namibia also has a development framework called Vision 2030, with HiAP being the adopted strategy. Kenya’s Vision 2030 can also play the same role, as all ministries report their performance to the Vision 2030 Secretariat. However, current collaboration for HiAP between government, development partners and civil society for HiAP is minimal. Strengthening these collaborations will foster HiAP progress towards implementation. WHO AFRO in 2013 [29] reported an intersectoral case study that addresses mental health policy reforms in Kenya, which was a United Kingdom government-funded project involving a range of stakeholders from child protection and social welfare, the Kenyan police and prisons, academia and an NGO-supported community engagement. Their collaboration brought together technical expertise and funds for mental health policy in Kenya [29, 30].

HiAP has received the highest political commitment globally, with the various WHO secretary generals endorsing it. Kickbusch et al. [31] highlighted examples of HiAP in Africa from South Sudan, Namibia and Zambia. All three have high political commitment for HiAP with heads of state acting as one of the policy entrepreneurs and officially committing to implementing HiAP in their countries. In Kenya, HiAP is in the policy document, but Ministers of Health after Hon. Charity Ngilu did not propagate HiAP as an agenda with the same focus and tenacity. The lack of commitment at the highest level – beyond the Ministry of Health – did not secure the HiAP approach as a political priority. Policies addressing HIV in Kenya represent a good example – once the president had declared it a national disaster, there was conscious action on it from all the other ministries [32, 33]. Establishing a ‘super department’ or ‘super committee’ with a HiAP strategy is essential [34]. Currently, there is an intergovernmental committee in Kenya, called the Apex, of which all ministers and governors are members and the president is chair; this can potentially be considered a super department.

A potential window of opportunity would be the budgeting process, as most interviewees indicated that ISC mostly happens at this time. This is when there can be a chance to evaluate each other’s policy objectives and can be an entry point for health considerations in the various policies. Around August, annually, all government departments receive advice on how to prepare their annual budget and allocated times for public participation. This allows contribution not only from the ministries but also from the public and civil society. The case for allocating a specific budget to HiAP could be made on such an occasion. The budgeting process and involvement of the Ministry of Finance is crucial for HiAP in Kenya, as most interviewees indicated that it is at budgeting time that they always review each other’s policies and plans for the given period. This might also send a signal to development partners willing to dedicate funds to implementing this approach. County health directors and the finance departments that are being set up at county level should also be able to consider HiAP in their budgeting process.

The World Health Assembly resolution WHA67.12 positioned HiAP as an essential strategy in addressing development in the post-2015 agenda. HiAP clearly promotes policy coherence among the various sectors. The post-2015 agenda, as a global development policy framework, enhances partnership among all stakeholders committed to the SDGs and allows the integration of HiAP [35, 36]. South Sudan, Namibia and Zambia have already committed to addressing SDGs with HiAP at the centre stage [31]. The Ministry of Planning and Devolution in Kenya is mandated to ensure that the SDGs have been understood throughout the decentralised governance system. County governments in Kenya have institutionalised SDG coordination through the directors of planning and economic affairs. SDGs have been decentralised to county level in the County Integrated Development Plan guidelines and in the County of Governors committee work plans [37]. This process can be a great facilitator for the nationwide spread of HiAP and create a win-win situation across sectors. However, its realisation will require a seamless implementation procedure, e.g. the Vision 2030 Medium Term Plan 3 could not fully incorporate goals from the African Union’s Agenda 2063 as well as the SDGs because of incompatibilities of timelines and indicators, according to a SDG Kenya Forum study [38]. This recent window of opportunity calls for further investigation for Kenya and has been taken up by the first author of this paper.

A study focusing on South Africa showed some similar barriers and facilitators for ISC for health [39], wherein lack of communication between sectors was the main issue; each sector perceived a lack of support from the others [39]. Another barrier was that ISC is a life-long issue whereas the tenure of policy-makers is finite, posing a challenge of commitment and involvement [39]. Regionally, global policies and institutions still have too much influence on countries, sometimes overlooking country priorities, and as such hampering national ownership [40]. Another barrier is that the main individuals who were involved in SDH and HiAP research in Kenya were either from academia or independent consultants; they were aware of global discourses, but this information did not necessarily transcend to policy-makers and civil society. As such, HiAP, like many polices, remains a primary global priority but is still gaining momentum as a national priority. The main way to foster national ownership for any intersectoral action in any country in the region is likely to start with political will and leadership from the highest level of government [34]. This should be coupled with effective engagement and coordination of all sectors at both the technical and operational levels [34, 41].

The literature on health policy processes in LMICs is relatively limited and seldom makes use of public policy frameworks [42]. Kingdon’s framework has been used in LMICs to study the emergence of a variety of health policies in sub-Saharan Africa. It has also been successfully used to evaluate how the Supreme Council of Health and Food Security in Iran promotes the government’s political commitment for HiAP from the national level to district level [43]. In the Iranian study, the setting is similar to Kenya, as the government is also decentralised and HiAP appears in the health policy document. However, unlike Kenya, Iran already has a ‘super department’, i.e. the Supreme Council of Health and Food Security, which is chaired by the president and constitutes leaders from the national level of government to the district level [43].

Using Kingdon’s framework to study the adoption of HiAP in Kenya was helpful. In particular, the dimensions of problems, policies and politics helped to answer how HiAP rose to the political agenda. It also helped to show to what extent policy actors adopted the HiAP approach in Kenya. It enabled us to identify several national policy entrepreneurs, such as Hon. Charity Ngilu; she played a critical role in pushing this agenda forward, despite a not entirely favourable politics stream. The main concern was that she did not remain in office long enough to prompt a sustained commitment at the highest political level. Yet, the study found that the importance of external actors’ influence – donors and international partners – on health-related agenda-setting processes was, to a large extent, eluded from Kingdon’s framework [12]. This is probably due to the fact that Kingdon’s framework was developed in a high-income setting, where international donors and organisations play little or no influence [12]. Researchers from LMICs should make sure to pay attention to this gap when applying this public policy framework.

This study has a number of limitations. We used a cross-sectional study design as a prospective approach could not be employed given the time and financial constraints. As many interviewees outside the health sector were also not aware of HiAP, they were not asked questions on the relevance of HiAP. Lastly, funding and resource allocation for HiAP in LMICs was an interesting aspect that merits an entire study on its own. It can be considered for future research.

## Conclusion

HiAP in Kenya is still in the early adoption and formulation stages. It has been endorsed in the policy documents, but great efforts need to be in place for HiAP to be implemented any time soon. The efforts include political will and leadership from the highest political office, which would foster national ownership and ensure a win-win situation across all government sectors. Issues relating to funding for the implementation of HiAP have to be considered from the onset so as to avoid it being a hindrance. Training and sensitisation of HiAP at the local level can be done by the linking of HiAP with SDG implementation under the Vision 2030 framework. Findings of this study are highly policy-relevant to decision-makers and programme implementers in other countries seeking to adopt HiAP in low-resource settings.

## Abbreviations

HiAP: Health in All Policies
ISC: intersectoral collaboration
LMICs: low- and middle-income countries
MDGs: Millennium Development Goals
SDGs: Sustainable Development Goals
SDH: social determinants of health
WHO AFRO: WHO Regional Office for Africa
